# *Saprolegnia parasitica* S1 and S2 Strains Differ in Zoospore Transition Timing and Pathogenicity Against Juvenile Atlantic Salmon ( *Salmo salar* )

**DOI:** 10.1111/jfd.70028

**Published:** 2025-07-17

**Authors:** James Duston, Mohammad Nasif Sarowar, Tyler Schmidt‐Schaun, Tessema Astatkie

**Affiliations:** ^1^ Faculty of Agriculture, Dalhousie University Bible Hill Nova Scotia Canada; ^2^ Onda Souris Prince Edward Island Canada

**Keywords:** ami momi, fungus, infection, oomycete, saprolegniasis, stress

## Abstract

S1 and S2 strains of *S. parasitica* are both common among diseased farmed salmonids in Nova Scotia, whereas globally S1 is rare and S2 is common. Following the initiation of asexual maturation and overnight incubation at 20°C then harvest, and incubation at 10°C in vitro, S2 secondary zoospores mostly transformed into cysts within 3 h, and by 6 h post‐harvest > 80% had germinated. S1 zoospores, by contrast, exhibited persistent motility; at > 30 h post‐harvest > 80% were in the motile stage, and < 10% were either cysts or germlings. The disease challenge test began with skin disruption by shaking pairs of fish in a net for 15 s, then stocking six fish in up to 12 aquaria (27 L water), addition of 400 zoospores/ml, held in static water for 11 h, then 10°C flow‐through. Through smolting (January–June) at 10°C, S1 was consistently more virulent than S2 (*p* = 0.005), with gross disease signs evident among 50% of test fish in 40 h for S1 versus 72 h for S2. Susceptibility to disease was independent of smolt status (*p* = 0.512). Secondary motile zoospores, cysts, or germling stages of both strains all caused disease, but S2 germlings were the least pathogenic.

## 1 Introduction

Saprolegniosis afflicts freshwater fish globally and is a costly burden to salmonid hatcheries (Magray et al. 2019). *Saprolegnia parasitica* , the principal pathogen, has a 63 Mb genome and a complex array of strains and phenotypic variability (Diéguez‐Uribeondo et al. 2007; Jiang et al. 2013). In Nova Scotia hatcheries, where the disease threat has increased markedly over the past decade, four strains were identified (Sarowar et al. 2019). The two most common, we named S1 and S2, were present in 37 and 42% of isolates from diseased fish, mostly salmonids. They differ by a single mutation of the internal transcribed spacer (ITS) region of the nuclear ribosomal DNA (nrDNA; Sarowar et al. 2019). S2 is common globally. Among Scottish salmon hatcheries, S2 was isolated from > 90% of fish suffering from saprolegniosis (Shreves et al. 2024). Moreover, the ITS sequence of S2 is identical to reference strain SAP208, which, in turn, matched 88 strains from around the world (Diéguez‐Uribeondo et al. 2007). S1, aside from Nova Scotia, has only been reported among brown trout ( *Salmo trutta* ) in Switzerland (Ravasi et al. 2018), and was absent from farms in Scotland (Shreves et al. 2024). S1 produced sexual structures in vitro at 18°C, a trait typical of non‐pathogenic saprophytic species within the *Saprolegnia* complex, whereas S2 did not, typical of pathogenic strains (Sarowar et al. 2019). The present study, by confirming S1 is pathogenic, is further evidence of the limitations of linking morphotype to pathogenicity among *Saprolegnia* spp. established by Willoughby (1978) and thoroughly reviewed by Diéguez‐Uribeondo et al. (2007).

Improving disease management has been slowed by difficulties inducing the disease under controlled conditions and identifying the infective stage within the complex asexual reproduction process. Asexual reproduction begins with sporangia releasing biflagellate primary zoospores which swim for 5–10 min then become primary cysts; neither pose a disease threat (Crump and Branton 1966). From primary cysts emerge faster swimming biflagellate secondary zoospores which form secondary cysts; both posited as infective agents. Intuitively, the duration of the motile secondary zoospore phase is important for seeking a host, especially since secondary cysts can ‘re‐mobilise’ up to six times, a process called polyplanetism (Die‐guez‐Uribeondo et al. 1994). Yet evidence is lacking, and their dispersion due to swimming was considered negligible compared to water column turbulence (Hallett and Dick 1981). The secondary cyst stage as the primary infective agent is also tenable; their long hairs with hooks seemingly ideal for latching onto the host (Pickering et al. 1979; Beakes 1983; Rezinciuc et al. 2018). Typically, strains with hooked hairs are pathogenic and hook‐less strains are saprophytes, but exceptions suggest the link is not causal (Fregeneda Grandes et al. 2001; Stueland et al. 2005). Secondary cysts germinate at some point, the emergent single hypha grows quickly and branches, forming a grey‐white coating visible on infected fish. Germlings might also serve as infective agents, but the evidence is unclear (Willoughby et al. 1983; Wood et al. 1988). Here, the results indicate motile secondary zoospores, cysts and germlings can all cause disease.

Healthy fish are resistant to saprolegniosis, requiring infection studies to include a stressor, such as skin abrasion, thermal shock, or a cortisol implant (Howe et al. 1998; Pottinger and Day 1999; Misk et al. 2022). Using a shortened version of ‘ami‐momi’ or ‘net‐massage’ pioneered by Hatai and Hoshiai (1993), we tested the hypothesis that smoltification increases the susceptibility to saprolegniosis (e.g., Pickering 1994). A causal link between the two has never been established despite smolting being associated with elevated blood cortisol titres, decreased skin mucus cell density and immune competence (Maule et al. 1987; Carey and McCormick 1998; O'Byrne‐Ring et al. 2003).

## 2 Materials and Methods

All procedures were approved by the Dalhousie University Faculty of Agriculture Animal Care and Use Committee (Files 1022666, 1036202, 1039484).

### 2.1 *S. parasitica* Culture

Single spore pure cultures of S1 and S2 were maintained on potato dextrose agar at 4°C (Sarowar et al. 2019). Pea broth, used to initiate asexual reproduction, was made by autoclaving frozen garden peas (62 g) in ca. 300 mL dH_2_O for 15 min (120°C). The pea juice was squeezed out through cheesecloth, topped up to 500 mL with dH_2_O, then autoclaved again and stored at room temperature. The 4‐day process, conducted in a biosafety cabinet (Microzone BK‐2‐4) at room temperature (ca. 20°C), began by inoculating up to 50 Petri dishes (10 cm) each with three agar plugs (each ca. 5 mm square) colonised with mycelia, then adding 25 mL of sterile pea broth. After 3 days, the mycelia typically covered > 75% of the Petri dish surface. On Day 3, starting at 17:00 h, to initiate asexual reproduction, the pea broth was drained off from each Petri dish, retaining the mycelial sheet. To each Petri dish, about 25 mL autoclaved dH_2_O at 20°C was added, then vigorously manually shaken for 30 s, then drained off. This washing, shaking, and draining sequence was repeated three times. Autoclaved fish tank water (ca. 20 mL) was then added, and the Petri dishes incubated overnight at 20°C. The next morning (start 07:30 h), the contents of each Petri dish were quickly agitated manually (20 s) with a cell scraper, then emptied into a plastic kitchen strainer (7 cm diam, 1 mm mesh). The filtrate drained into a glass beaker sitting on a small bed of ice, cooling it from 20°C to 10°C in about 1 h, to match the fish rearing temperature. Immediately post‐harvest, the relative abundance of motile secondary zoospores, cysts, and germlings was quantified using a haemocytometer (Brightline, Reichert‐Hausser, NY). The average sum of the three stages of spores was ca. 5 × 10^6^ per Petri dish. The zoospore suspension was stored at 10°C in the glass beaker covered with aluminium foil, and always stirred gently but thoroughly before decanting or sampling.

### 2.2 Transition Between Motile, Cyst, and Germling Zoospore Stages

The timing of transition between the three zoospore stages in vitro among the two strains was compared in a series of nine tests between November 2021 and June 2022. In each test, each of the two strains was harvested, and the filtrate was incubated in a 500 mL glass beaker in a water bath at 10°C. Two samples (each 20 μL) from each beaker were taken at nine intervals up to 48 h post‐harvest. For each sample, each of the three zoospore stages was counted in each of eight squares (0.1 mm^3^) of a haemocytometer and the relative abundance expressed as a percentage.

### 2.3 Disease Challenge Tests

Atlantic salmon parr (*n* = 500; Saint John River strain; age 0+) from a commercial hatchery (Cooke Aquaculture, Millbrook Hatchery, Truro, NS) were received in October 2021. They were reared in a green fibreglass tank (1500 L) with a flow‐through supply of 10°C freshwater from a deep well (pH 8.0; alkalinity 111, hardness 210 mg/L CaCO_3_ eqv.) under a simulated natural photoperiod cycle (Latitude 45^o^N) and fed a commercial diet (Skretting). The mean (SE) fork length and body weight in December were 15.3 (0.14) cm, 46.4 (1.3) g, and by June they were 18.4 (0.11) cm and 68.5 (1.6) g, respectively. Condition factor (mean, SE) decreased during smolting from 1.27 (0.013) in December to 1.05 (0.010) in late April, then increased to 1.10 (0.018) in early June, indicating the transition to post‐smolt.

Food was withheld for 3 days prior to a test. Fish selection began at 07:00 h, taking two people about 20 min. Fish were caught with a knotless dipnet (40 × 40 cm; 5 mm mesh; Dynamic Aqua Supply, Surrey, BC), then transferred to a bucket (25 L) half filled with rearing tank water, then gently poured into an insulated holding bin (250 L; Xactics, Cornwall, ON) containing about 100 L of rearing tank water aerated by a silica diffuser. Using a small aquarium dipnet (15 × 10cm), up to three fish at a time were anaesthetised (ca. 10 L, 0.1 g/L MS222 ‘Syncaine’ Syndel, Nanaimo, BC). At a loss of reflex, the fish were transferred by net into a shallow bath of recovery freshwater, then each was picked up with an ungloved hand and examined, male mature parr were rejected. Fish were then transferred to a recovery bin (ca. 150 L 10°C water, silica diffuser, 250 L Xactics), lid partially on, and allowed about 1 h recovery while the zoospores were harvested.

Challenge tests were conducted in an isolated lab with 12 plexiglass tanks (30 × 75 × 45cm deep, stand‐pipe water depth 16 cm; 36 L), supplied with the same 10°C ground water as the larger rearing tank. Dissolved oxygen was maintained at > 90% saturation via a diffuser supplied with oxygen. Light intensity at tank level was < 50 lx, the source a fluorescent ceiling light dimmed with a filter. Photoperiod was LD24:0. About 1 h before the tanks were stocked with fish, the water supply was turned off, and the level lowered to 12 cm (volume = 27 L) to reduce the quantity of zoospores needed. Twelve fish, one at a time, were netted at random from the Xactics is recovery tank into a large white bucket (30 L, 29 cm diameter) about 1/3 full of water and carried (1 min) to the challenge lab. For ami‐momi, two fish were caught in a small dipnet (15 × 20 cm; Dynamic Aqua Supply Ltd., Surrey BC), shaken in air for 15 s in a horizontal plane (amplitude ca. 10 cm, frequency ca. 1 s), then released into a tank. Each tank was stocked with two fish in sequence until there were six fish per tank. *S. parasitica* treatment was assigned randomly to each tank. Two tanks per treatment.

Preliminary tests indicated a test dose of 400 zoospores/mL of S2 was suitable. Doses 100 to 300/mL took about 7 days to reach 70%–80% diseased fish, followed by several days of uncertainty waiting for the asymptote. Doses > 500/mL resulted in 100% diseased fish in a similar time‐frame to 400/mL, but required extra effort to produce sufficient zoospores. Negative controls receiving zero zoospores exhibited zero disease signs, hence were not included in the main tests. Also, fish showed no interest in food during preliminary trials, so none was offered during the main tests.

To start a challenge test, the volume of harvested zoospores to achieve 400 zoospores/ml was measured in a glass volumetric cylinder (100 mL), then poured into the assigned tank and stirred thoroughly for about 5 s using the empty measuring cylinder. By 09:00 h inoculation was complete. The tank water remained static for 11 h, during which it was warmed from 10°C to about 14°C due to ambient air temperature. Unionised ammonia after 11 h was consistently < 0.001 mg/L. The water supply to each tank was then turned on with a flow‐through rate of 8 L/min. Disease signs, recorded at ca. 4 h intervals, included skin discoloration, mycelia on skin and fins, and abnormal swimming behaviour. At loss of equilibrium, an individual fish was netted out and euthanised (MS222 overdose); fork length, body weight, and sex were recorded. At the test end‐point, when % diseased fish had reached an asymptote, survivors were euthanised, tanks drained, and disinfected.

### 2.4 Pathogenicity of S1 and S2 Through the Parr‐Smolt Transformation

Five tests were conducted over 6 months (December 24, January 22, February 25, April 29, June 3). For each test, four tanks were each stocked with six fish. Two tanks were each inoculated with either S1 or S2 zoospores, freshly harvested. The dose was 400 zoospores per mL except for the April 29 test when it was 100/mL due to a poor yield. Within each test, the initial dose of S1 and S2 zoospores per ml was the same, but the proportion of motile zoospores, cysts, and germlings differed.

### 2.5 Effect of *S. parasitica* Strain and Zoospore Development Stage on Disease

In four disease challenge tests repeated between January and June, the effect of strain (S1 vs. S2) and zoospore developmental stage was explored by exposing salmon to inoculants that were either freshly harvested (0 h) or 11 h post‐harvest after holding cultures in vitro at 10°C. S1 cultures at 0 and 11 h post‐harvest were dominated by motile secondary zoospores: their mean proportion increased between 0 and 11 h post‐harvest from 64% to 85% (Table 1). S2 zoospores, by contrast, transitioned from 74% cysts to 94% germlings between 0 and 11 h (Table 1). The test procedure and dose (400 zoospores/mL) were the same as described in Section 2.3, with the addition of a second group of fish subjected to ami‐momi in the evening, the inoculants added around 20:00 h at 11 h post‐harvest, and the tank water remaining static until 07:00 h the next day.

**TABLE 1:** Mean (SE) percent of each zoospore stage of S1 and S2 strains of *Saprolegnia parasitica* either freshly harvested (0 h) or 11 h post‐harvest following incubation in vitro in autoclaved fish tank water at 10°C. The disease response of juvenile Atlantic salmon is summarised by the fitted parameter estimates of logistic growth regression model. Parameters sharing the same letter are not significantly different at the 5% level. Data are derived from four tests run between January and June, 2022.

Strain	S1		S2	
Zoospore hours post‐harvest	0 h	11 h	0 h	11 h
Motile %	64 (3.2)	85 (0.7)	7 (2.5)	1 (0.36)
Cyst %	35 (3.5)	8 (2.7)	74 (6.3)	5 (3.0)
Germling %	1 (0.5)	7 (2.5)	19 (6.3)	94 (3.4)
Theta 1 (ϑ_1_) Asymptote (% diseased fish)	94.7^c^	97.4^b^	100^a^	89.0^d^
Theta 2 (ϑ_2_) Days to 50% of ϑ_1_	2.3^d^	2.9^c^	3.3^b^	4.7^a^
Theta 3 (ϑ_3_) Days between ϑ_2_ and ca. 3/4 ϑ_1_	0.502^a^	0.539^a^	0.633^a^	0.648^a^

### 2.6 Statistical Methods

For the analysis of the transition between zoospore stages, the single experimental factor was *S. parasitica* strain (two levels: S1 or S2). Between each strain, the mean proportion (%) of each of the three zoospore stages (motile, cyst, germling) was compared at six of the nine sampling intervals (0, 4, 5, 12, 24, and 48 h) where there were sufficient values to be compared using a 2‐sample *t*‐test with equal or different variances. For each variable and each time interval, the normal distribution assumption on the error terms was verified using the normal probability plot of residuals and the Anderson‐Darling test for normality (Montgomery 2020). For S1, the relationship between hours post‐harvest and the proportion of both motile zoospores and cysts was best described by a second‐order polynomial ($Y=\beta_{0}+\beta_{1}X+\beta_{2}X^{2}+\varepsilon$). For S2, by contrast, the relationship between hours post‐harvest and the proportion of both motile zoospores and cysts was best described by an exponential decay (nonlinear) regression model ($Y=\theta_{1}e^{\theta_{2}X}+\varepsilon$). The progressive increase in the proportion of the germling stage among both S1 and S2 was best described by the asymptotic (nonlinear) regression model ($Y=\theta_{1}-\theta_{2}e^{-\theta_{3}X}+\varepsilon$). The parameters were estimated iteratively as described in Bates and Watts (1988).

The disease response through the parr‐smolt transformation was analysed using the general linear model. The design was a two‐factor factorial; the factors were time (five levels: December 21; January 22; February 22; April 29; June 3), and *S. parasitica* strain (two levels: S1 and S2). The normal distribution and constant variance assumptions on the error terms were verified by examining the residuals (Montgomery 2020).

Data from the four tests comparing strain (two levels: S1, S2) and zoospore developmental stage (two levels: freshly harvested, 11 h post‐harvest) were pooled since the disease response was independent of smoltification. The relationship between % diseased fish (Y) and days post‐exposure (X) almost perfectly fits the Logistic Growth model:

$$\left(Y=\frac{\theta_{1}}{\left(1+e^{\left(-\frac{\left(X-\theta_{2}\right)}{\theta_{3}}\right)}\right)}+\varepsilon \right)$$

$\theta_{1}$ is the asymptote (the highest value of Y), $\theta_{2}$, the value of X where Y reaches 50% of $\theta_{1}$, and $\theta_{3}$, the interval in X between $\theta_{2}$ and about 3/4 of $\theta_{1}$. The model was modified by adding incremental parameters to check whether the parameters of the four combinations were significantly different or not. The statistical analysis was completed using Minitab software.

To allow comparison between published papers of the base pair position of the mutation in the ITS region between *S. parasitica* strains, the sequences were retrieved from Genbank and then aligned using https://mafft.cbrc.jp/alignment/server/index.html.

## 3 Results

### 3.1 Transition Between Motile, Cyst, and Germling Zoospore Stages

**FIGURE 1:**
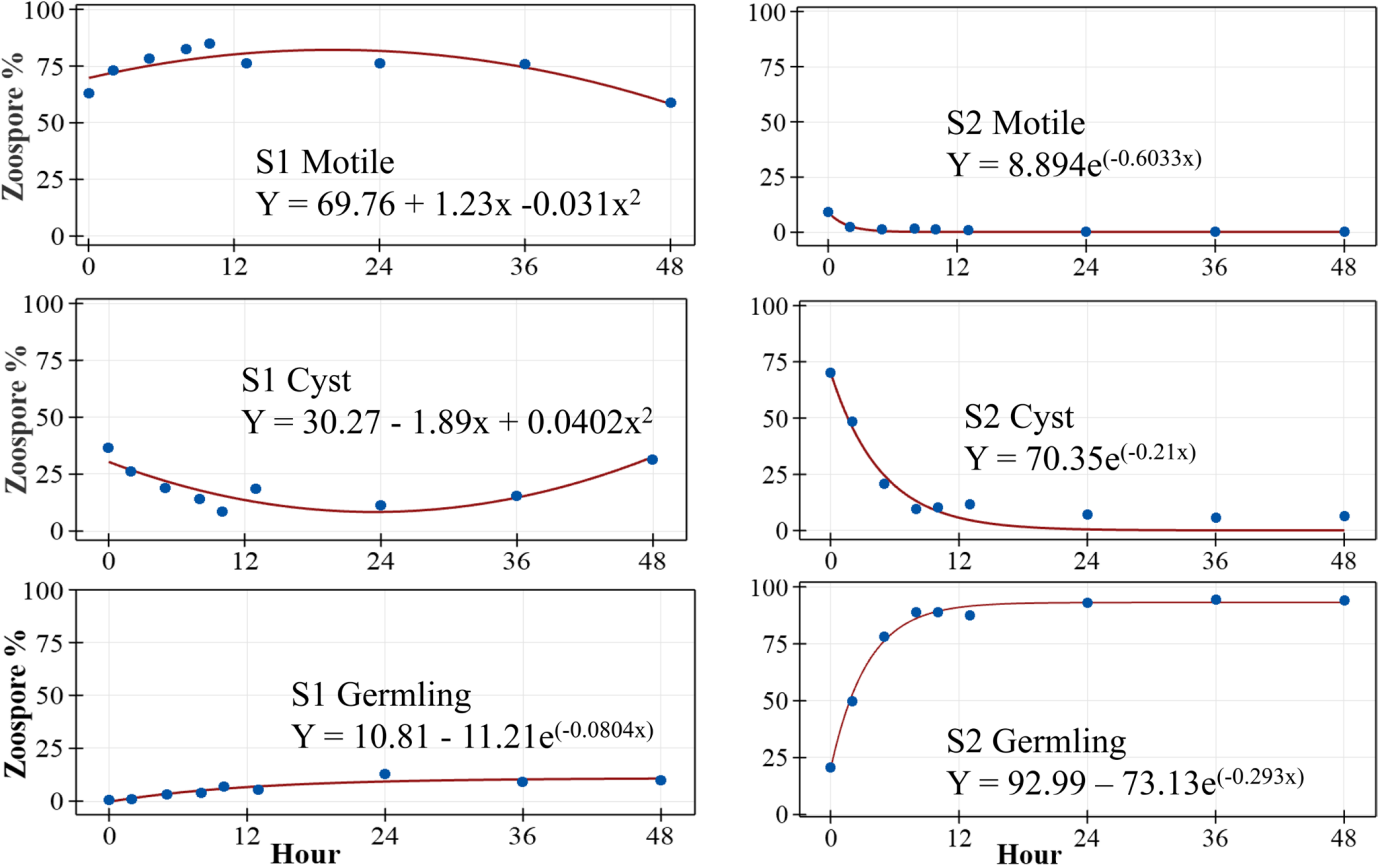
Relationship between the percentage of motile secondary zoospores (upper panel), cysts (middle panel), and germlings (lower panel) of S1 and S2 strains of *Saprolegnia parasitica* and hours following harvest and incubation in vitro at 10°C. Asexual reproduction was initiated at 20°C, 14 h before harvest. In the fitted models, Y, proportion of zoospores and X, hour.

The transition from motile secondary zoospore to immobile cyst to germling stage was much faster among S2 compared to S1 following the initiation of asexual reproduction and overnight incubation in autoclaved fish rearing tank water. At 1 h post‐harvest, ca. 15 h post‐initiation, the proportion (%) of motile zoospores was seven‐fold lower among S2 compared to S1 (9 vs. 63%; *p* < 0.001; Table 2). Between four and 48 h post‐harvest, the proportion of motile zoospores of S2 further decreased to < 2%, whereas S1 remained at 60 to 80%, significantly higher (*p* < 0.001; Table 2; Figure 1).

**TABLE 2:** Comparison between the mean % proportion of motile, cyst and germling zoospore stage of S1 and S2 strains of *Saprolegnia parasitica* incubated in vitro at 10°C at selected time intervals up to 48 h. *p*‐values were derived from 2‐sample t‐test to compare the mean values of each strain.

Zoospore stage	Hour	S1 mean	S2 mean	*p*
Motile %	0	63.2	9.0	< 0.001
	4	75.4	2.0	< 0.001
	12	79.8	1.1	< 0.001
	24	72.9	0.2	< 0.001
	48	58.8	0.0	NA
Cyst %	0	36.4	70.3	< 0.001
	4	22.7	23.4	0.943
	12	14.9	11.3	0.525
	24	12.4	7.6	0.348
	48	31.3	6.3	0.027
Germling %	0	0.4	20.7	< 0.001
	4	1.8	74.6	< 0.001
	12	5.3	97.6	< 0.001
	24	14.7	92.2	< 0.001
	48	9.9	93.7	< 0.001

Abbreviation: NA, not available because all values of S2 were 0.

The proportion of cysts at harvest was 70% among S2, almost two‐fold higher than S1, 36% (*p* < 0.001; Table 2). S2 cyst proportion decayed exponentially to 23% at 4 h post‐harvest, 11% at 12 h, and 6% at 48 h (half‐life 3.3 h; Figure 1). S1 cyst proportion, by comparison, remained more stable over the 48 h test period, decreasing from 22.7% at 4 h post‐harvest to 14.9% at 24 h, at which point the proportion of S1 cysts was similar to S2 (*p* = 0.348, Table 2; Figure 1). At 48 h post‐harvest, the mean proportion of S1 cysts was significantly greater than S2 (31.3 vs. 6%; *p* = 0.027; Figure 1).

The proportion of germlings among S2 increased rapidly from a mean of 20% at harvest to an asymptote of > 80% at 5 h post‐harvest (Figure 1). S1 germling mean proportion also increased towards an asymptote, but remained < 15% throughout, highly significantly lower than S2 at all sampling intervals (*p* < 0.001; Figure 1; Table 2).

### 3.2 Pathogenicity of S1 and S2 Through the Parr‐Smolt Transformation

**FIGURE 2:**
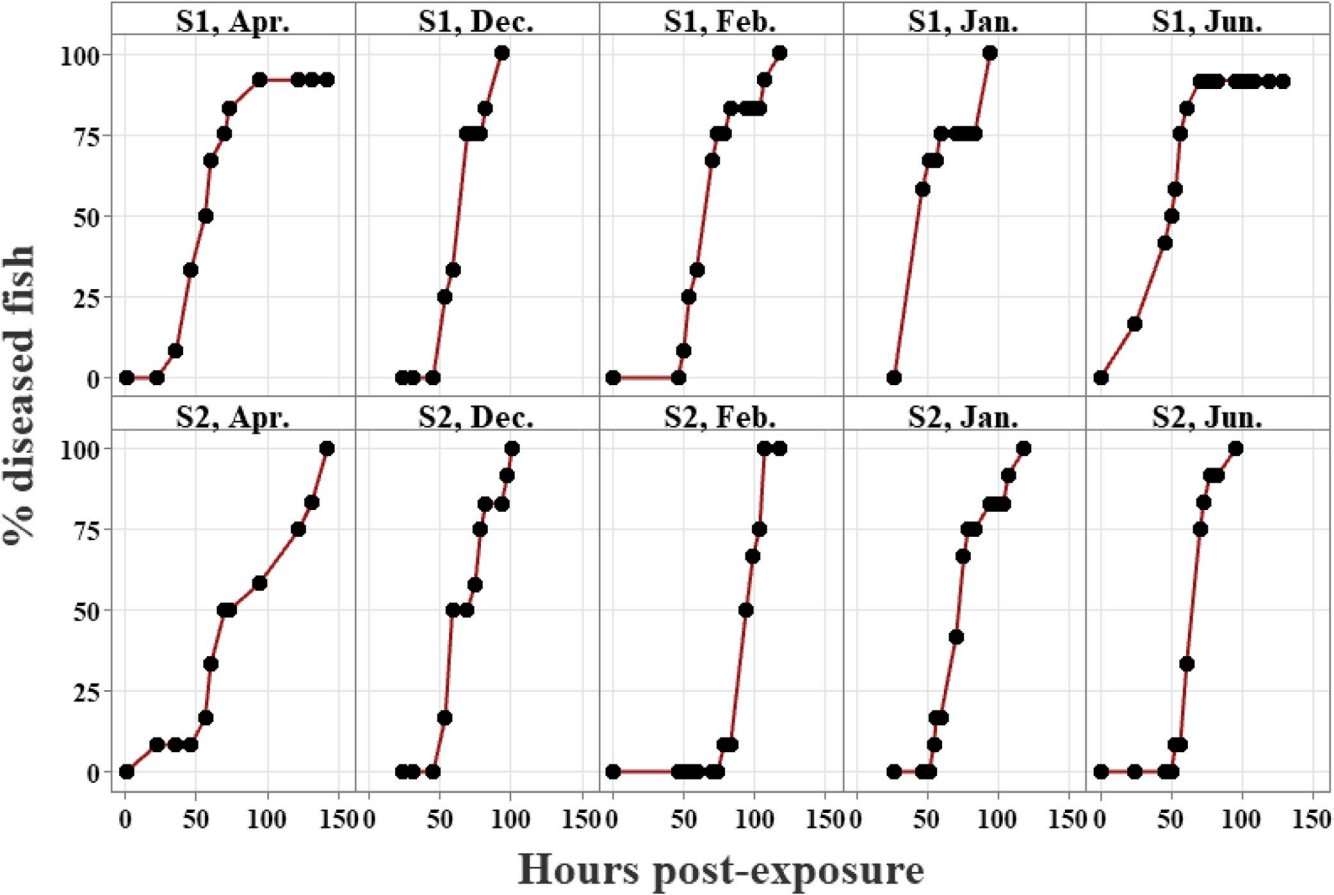
Disease challenge test response of Atlantic salmon through the parr‐smolt transformation (Dec. 24; Jan. 22; Feb. 25; Apr. 29; Jun. 3)) exposed to *Saprolegnia parasitica* strain S1 (upper panel) or S2 (lower panel). *N* = 12 fish per test. The initial zoospore dose was 400/mL except for the April test, which used 100/mL due to a low yield. Rearing water was static for 11 h, then flow‐through well water (10°C). End‐point: When an individual diseased fish lost equilibrium, it was removed and euthanised.

In all five diseases challenge tests between December and June, exposing the salmon to freshly harvested zoospores of either S1 or S2 strain resulted in > 90% disease incidence in about 4 days (Figure 2). S1 was very significantly more virulent than S2 (*p* = 0.005). The strain effect was most evident in tests 2 and 3 where the estimated time to 50% disease incidence for S1 was 40 and 65 h compared to 72 and 94 h respectively for fish exposed to S2 (Figure 2). The transition from parr to smolt had no significant effect on the disease response (*p* = 0.512; Figure 2). The interaction between strain and the date (month) of the test was not significant (*p* = 0.431). The disease response to the April 29 test that used a low dose (100 zoospores/mL) for S1 was similar to the other four tests, but for S2 had a slower progression (Figure 2).

Disease signs began with mycelia visible on fin tips and skin, then abnormal swimming behaviour, the body at a 45° angle, head up, which progressed to loss of equilibrium at which point a fish was removed and euthanised. Post‐mortem, abnormalities commonly observed included mottled discolouration of the skin, fin erosion and haemorrhage of the lower canthus of the eye. Gills were consistently not infected.

### 3.3 Effect of Strain and Zoospore Development Stage on Disease

**FIGURE 3:**
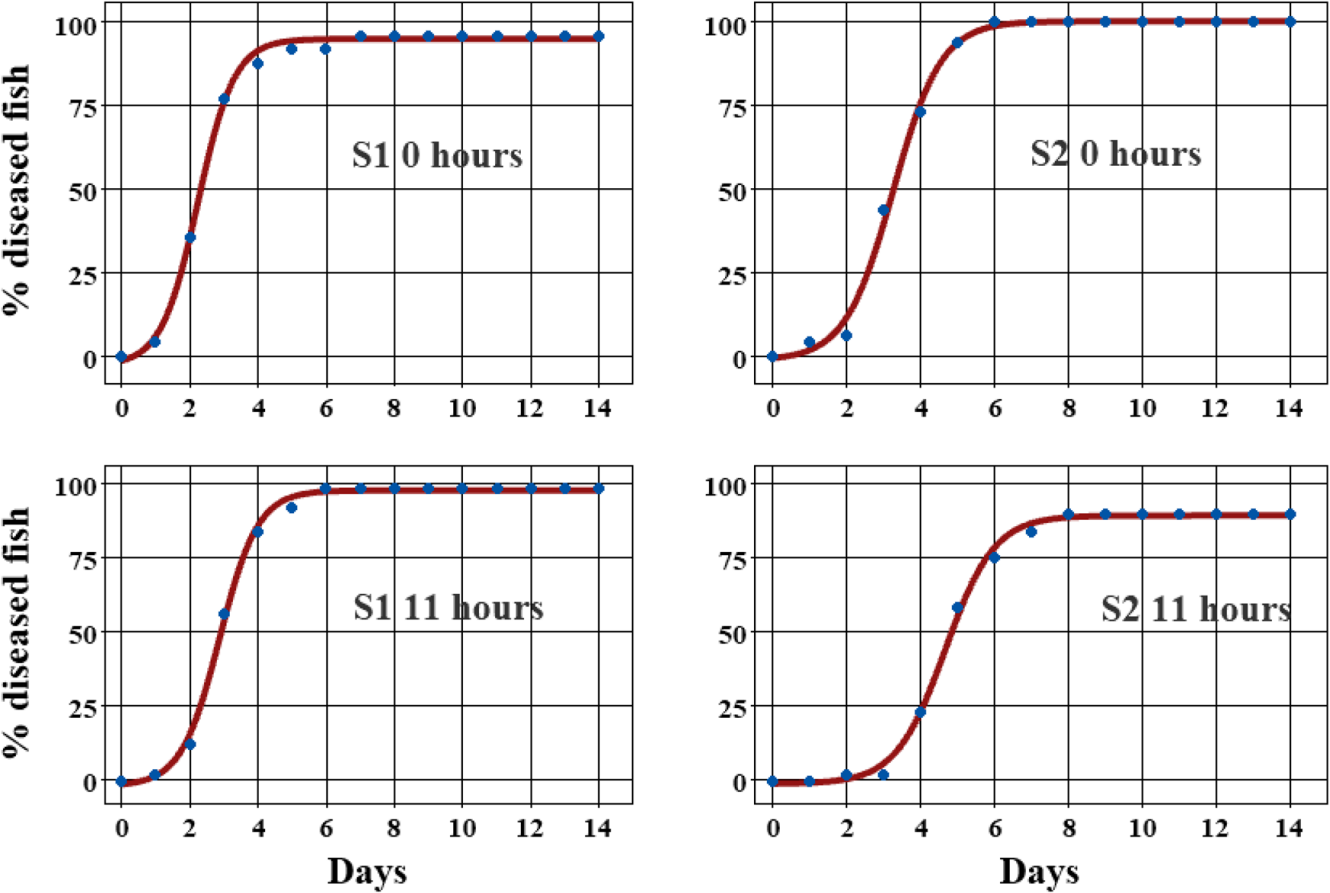
Fitted logistic growth regression models for the disease response of juvenile Atlantic salmon at 10°C following exposure to either S1 or S2 strain of *Saprolegnia parasitica* zoospores that were either freshly harvested (0 h) or 11 h post‐harvest held in vitro at 10°C. Coordinates are mean values from four challenge tests (start: 22 January, 25 February, 29 April, 3 June). Within each test, each of the four treatments was composed of *n* = 12 fish divided between two aquaria. The proportion of each zoospore stage at inoculation is shown in Table 1.

All treatments caused disease, and the effect of both strain and zoospore development stage was significant. The response was consistent among the four tests run between January and June, allowing the data to be pooled and described by a logistic growth curve (Figure 3). Theta 1(θ_1_), the asymptote of % diseased fish, was reached in 6–8 days post‐inoculation and ranged from 89.0% to 100% (Table 1; Figure 3). Judged by this parameter, the least pathogenic treatment (θ_1_ = 89%) was S2 11 h post‐harvest, which comprised 94% germlings (Table 1). The most pathogenic treatment was freshly harvested S2 composed of 74% cysts at inoculation, θ_1_ = 100%. S1 strain, either freshly harvested or 11 h post‐harvest, was composed of 64% and 85% motile secondary zoospores at inoculation and reached an asymptote of 94.7% and 97.4% (Table 1).

Theta 2 (θ_2_), the days to 50% of the asymptote, an indicator of the speed of disease progression in susceptible fish, also differed significantly between all four treatments. The least pathogenic treatment was, again, S2 11 h post‐harvest with 94% germlings, reaching θ_2_ in 4.7 days. The most pathogenic treatment was S1 freshly harvested (S1 0h), θ_2_ = 2.3 days, closely followed by S1 (11 h), θ_2_ = 2.9 days (Table 1).

## 4 Discussion

Identifying a significant difference in pathogenicity between two strains *S. parasitica* that vary by a single base pair in the ITS region of the nrDNA region is novel. The contrast between strains in the timing of transition between zoospore stages is further evidence of substantial phenotypic differences between S1 and S2, adding to their contrasting ability to produce sexual structures in vitro at room temperature (Sarowar et al. 2019). Our attempt to address two long‐standing hypotheses indicated that motile, cyst, and germling stages can all cause disease, and that saprolegniosis risk is independent of the transformation from parr to smolt.

The challenge tests verified S1 is pathogenic, confirming this strain was the cause of saprolegniosis following its isolation from four species of diseased farmed salmonids (Sarowar et al. 2019). That S1 is significantly more pathogenic than S2 allows us to postulate this strain is responsible for the increasing problem of saprolegniosis in Nova Scotia. Whether S1 mutated from S2 here in Nova Scotia or was introduced is unknown. Mutation seems more likely since S1 has only been reported once, in Switzerland (Ravasi et al. 2018). A global survey of 128 isolates of the *Saprolegnia* spp. complex revealed 88 were identical to S2, which was named SAP208 in Dieguez‐Uribeondo et al. (2007). Eleven *S. parasitica* strains differed from S2 by up to four mutations in the ITS region, of which seven differed by a single base pair (Diéguez‐Uribeondo et al. 2007). The pathogenicity of these 11 strains relative to S2 is unknown. The single ITS mutation that distinguished S1 from S2 was reported at 630 bp by Sarowar et al. (2019) and 598 bp by Shreves et al. (2024). The numerical disparity is due to differences in the length of the DNA strand sequenced, a consequence of the quality of the extraction process. Aligning these sequences with the SAP208 reference strain indicates S1 was not detected by Diéguez‐Uribeondo et al. (2007). Using their counting scheme, the single S1 ITS mutation is at 649 bp, whereas the nearest mutations were at 632 and 699 bp in Diéguez‐Uribeondo et al. (2007). If analysis of the ITS region remains an essential tool, then researchers need to agree on a standardised bp counting method. However, its utility may be limited since four *S. parasitica* isolates sharing the same ITS sequence differed markedly at the whole genome level and exhibited differences in host preference and virulence (Matthews 2020; Matthews et al. 2021).

A contrast between *S. parasitica* strains in timing of the transition between zoospore stages was first reported by Willoughby et al. (1983), but since then has received little attention. The rapid germination by S2 described here was comparable to the ‘ready encystment and growth’ exhibited by 10 isolates originally classified as *S. diclina* Type 1, later re‐evaluated as *S. parasitica* (Willoughby et al. 1983; Diéguez‐Uribeondo et al. 2007). Most of these 10 isolates were from diseased salmonids, including ATCC 42062 from a brown trout which shared the same ITS sequence as S2 (Diéguez‐Uribeondo et al. 2007). The second group of 12 isolates identified by Willoughby et al. (1983) exhibited ‘persistent motility’ of the secondary zoospore stage comparable to S1. Only one of these isolates, C17, from a diseased Arctic charr ( *Salvelinus alpinus* ), was classified as a typical salmonid pathogen based on its morphology. Three others: SC, 776 and 800 were isolated from diseased non‐salmonids *Perca fluviatilis* , *Cyprinus carpio* and *Tinca tinca* L., respectively, and the other eight were non‐pathogenic saprophytes (Willoughby et al. 1983). The persistent motility of secondary zoospores exhibited by C17 and SC might have been due to polyplanetism, suggested Willoughby et al. (1983), stating a high proportion of empty, ornamented zoospore cyst cases were detected using transmission electron microscopy (Pickering et al. 1979). Empirical evidence of polyplanetism in Pickering et al. (1979), however, is lacking; the prevalence of empty secondary cyst cases merely reported as “occasional”. Among the other strains exhibiting persistent motility of secondary zoospores, Willoughby et al. (1983) were highly confident it was independent of polyplanetism since empty cysts were never observed. Exploring the mechanism of persistent motility of S1 secondary zoospores was beyond the scope of our study. Certainly, persistent motility was associated with the higher pathogenicity of S1, but we refrain from suggesting a causal link since several non‐pathogenic saprophytes also displayed this characteristic (Willoughby et al. 1983). Among oomycete plant pathogens, by contrast, the biflagellate secondary zoospore stage is accepted as the infective agent, swimming through microfilms of static water towards host tactic signals at 15 mm min^−1^ or so (Appiah et al. 2005; Tran et al. 2022). *S. parasitica* motile zoospores also exhibited chemotaxis to a salmonid skin homogenate in vitro, supporting the hypothesis this stage is an infective agent (Matthews et al. 2021). In freshwater ecosystems, however, Hallett and Dick (1981) rightly pointed out active movement of oomycete zoospores is insignificant compared to natural turbulence. To increase the chances of physically making contact with a highly motile fish host, it seems a useful adaptation to include both cyst and germling stages as additional infective agents.

Disease following exposure of fish to the inoculant containing 94% germlings of S2 strain is the best evidence to date this stage of *S. parasitica* serves as an infective agent. Similarly, the accumulation of *S. parasitica* ‘propagules’ in the skin mucus of brown trout following the inoculation of tank water with strain ATCC 42062 (= S2) that germinated rapidly suggested germlings were an infective agent, in addition to encysted zoospores (Wood et al. 1988). Beakes et al. (1994) considered rapid germination a useful adaptation to help anchor the germling in the fish epithelium to reduce the risk of being sloughed‐off with the mucus. The cyst stage of *S. parasitica* has also received much attention as an infective agent; the long‐hooked hairs associated with a three‐fold greater adhesion strength compared to hair‐less saprophytes (Rezinciuc et al. 2018). Yet establishing a causal link has proved difficult and is upset by ‘outliers’ such as non‐pathogenic ‘Strain A’ with long, hooked hairs in bundles on the secondary cysts (Stueland et al. 2005).

A deficiency of the study was our ignorance of the fate of the zoospores during the 11 h static water phase post‐inoculation. Attempts to quantify zoospore density by observing 1 mL water samples in a Sedgewick‐Rafter cell under a phase‐contrast microscope failed. The only two previous studies to quantify propagules post‐inoculation used more elaborate procedures. Pottinger and Day (1999) counted germinated spores in a Sedgewick‐Rafter cell in a minimum of four samples following overnight incubation of 100 mL water samples in 250 mL flasks that included antibiotics. An earlier study in the same lab estimated the density of *S. parasitica* propagules by counting the number of colonies following 72 h incubation in Petri dishes containing a Polycell‐gel (Wood et al. 1988). Following a single inoculation, *S. parasitica* propagule density was 15–25 mL^−1^ for up to 24 h following a single inoculation (Wood et al. 1988), whereas a slow‐release device maintained 2–6 mL^−1^ for up to 11 days (Pottinger and Day 1999). Going forward, quantitative PCR may be useful (Rocchi et al. 2017), but microscopy would still be needed to determine the proportion of the three zoospore stages. Since Willoughby et al. (1983) showed zoospore development is dependent on the chemical composition of the incubation water, the fate of the spores in the test aquaria with live fish might differ from a beaker with sterilised fish water.

The ami momi procedure differs between studies, potentially altering the disease response and results interpretation. The shaking of two fish in a small net for 15 s used here was a marked contrast to 10–20 small fish in a large net shaken for 120 s in most previous studies (Table 3). Our approach was best suited to the relatively large body size of the test fish, ensuring each individual was equally exposed to the net face, and addressed ethical concerns. The fish suffered zero acute ‘shock’ mortality compared to 5%–13% in two other studies (Table 3). The test dose of 400 zoospores per ml was higher than previous studies, which ranged from 10 to 300 per mL (Table 3). Preliminary trials indicated doses as low as 25/mL could cause disease, but the response tended be slower and less predictable, as illustrated by the S2 test in April, when the zoospore yield was 100/mL (Figure 2). In previous studies, consistent disease responses were achieved with doses as low as 10 and 25 spores per mL (Table 3). However, strain ATCC 90213 (= S2), exposed to a low dose of 10 spores/mL resulted in a disease incidence of only 18% among 19 g *S. salar* parr in 16 days (Stueland et al. 2005), whereas a 20‐fold higher dose resulted in 100% mortality in 10 days among both 20–30 g coho and 20 g rainbow trout ( *Oncorhynchus mykiss* ; Hatai and Hoshiai 1993; Yuasa and Hatai 1995; Table 3). In the same study by Stueland et al. (2005), two strains isolated from parr in Norway and Scotland were markedly more pathogenic at 10 spores/mL, resulting in 31% and 89% mortality, respectively. Based on Shreves et al. (2024), there is a > 90% probability that the Scottish strain was S2, the same as ATCC 90213 from Japan. Why the same strain exhibited a five‐fold difference in pathogenicity between the trials in Japan vs. Norway is puzzling. The 11 h static water period in the present study was markedly shorter than previous studies, which ranged from 24 to 72 h (Table 3). Our initial trials using 24 h static water resulted in unacceptable warming to 18°C due to the indoor lab set‐up. An 11 h static water exposure limited warming to 14°C and facilitated exposing fish to zoospores at different developmental stages on the same day. In preliminary trials, static water periods of 2, 6 or 11h recorded a similar disease response among parr (unpublished data). Similarly, *S. parasitica* spores adhered within 2 h to the skin mucus of brown trout in a 65 L aquarium (Wood et al. 1988). Going forward, a shorter static water period would simplify the test and facilitate targeting test fish with spores of a known developmental stage. Given the economic importance of saprolegniosis to the salmon farming industry, the development of a standardised challenge test used globally would speed up advances in the state of knowledge and disease management.

**TABLE 3:** Comparison of methods used in *Saprolegnia parasitica* disease challenge tests on salmonids that included disrupting the skin by shaking fish in a dip‐net (‘ami momi’).

Study	Hatai and Hoshiai (1993); Yuasa and Hatai (1995)^b^	Fregeneda Grandes et al. (2001)	Stueland et al. (2005)	Matthews et al. (2021)	Misk et al. (2022)	Present study
Test species	*O. kisutch* , *O. mykiss* ^b^	*O. mykiss*	*S. salar*	*S. trutta*	*S. salar*	*S. salar*
Mean body weight (g)	25, 19^b^	19	21	1.2	43	57
Food withheld (days)	a few	3	3	0	1	3
Net mesh (mm)	5	6	5	^a^	^a^	1
Net dimensions (cm)	31.5 × 30	37 × 28 × 35	^a^	^a^	^a^	15 × 20
Number of fish shaken	10 or 15^b^	15	20	1	10	2
Shake duration (s)	120	120	120	30	120	15
Mucus rinsed off	Yes	Yes	Yes	^a^	^a^	No
Shock mortality (%)	13^b^	5	^a^	0	^a^	0
Dose (zoospores ml^−1^)	200	200 or 300	10	300	25	400
Static water hours	72	72	48	24	48	11
Static water vol. (L)	48 or 19^b^	27	200	1	^a^	27
Density (kg m^−3^)	5.1 or 14.8^b^	10.6	4.1	1.2	20	12.7
Temperature (°C)	12–13	10–12	9–10	12	10	10
Days to disease asymptote (approx.)	10	12	^a^	7	10–20	6

^a^ No information.

^b^ indicates the data from Yuasa and Hatai.

The independence between smoltification and susceptibility to saprolegniosis reported here is perhaps surprising given the increase in cortisol levels, immunosuppression and a decrease in epidermal mucus cells associated with the parr smolt transformation (Maule et al. 1987; Carey and McCormick 1998; O'Byrne‐Ring et al. 2003). Yet firm evidence of a causal link between smolting and saprolegniosis is lacking in the literature. Confounding factors among hatchery reared fish include stress associated with injection vaccination, seasonal increases in saprolegnia load (Beckmann et al. 2020; Waterstrat 1997). The duality of cortisol further complicates the picture; being both adaptive, stimulating the increase in salinity tolerance, and maladaptive, associated with immunosuppression (Breves et al. 2024; Maule et al. 1987). Some of our preliminary trials indicated smolts were more susceptible to disease than parr, but that result was not repeatable here. Possibly, the ami momi procedure was so stressful and damaging to the skin it obscured the comparatively subtle changes in skin morphology and immunity associated with smoltification.

In conclusion, the present study re‐emphasised the great value of the ami momi procedure to study saprolegniosis. Our version of the test yielded robust data with relatively few fish. That S1 was more pathogenic than S2, despite producing sexual structures in vitro, was further evidence of the limitations of the classical morphological criteria for defining the *Saprolegnia* spp. complex. Comparing the phenotype of strains that differ by a single mutation in the ITS region of the nuclear ribosomal DNA appears a useful way to study this important pathogen. When using this approach, information dissemination would be facilitated by a standardised base pair numbering system.

## Author Contributions

**James Duston:** funding, conceptualization, investigation, writing. **Mohammad Nasif Sarowar:** conceptualization, writing – review and editing. **Tyler Schmidt‐Schaun:** investigation, writing – review and editing. **Tessema Astatkie:** formal analysis.

## Conflicts of Interest

The authors declare no conflicts of interest.

## Data Availability

All data presented is original, none shared.
